# Glucocorticoids accelerate maturation of the heme pathway in fetal liver through effects on transcription and DNA methylation

**DOI:** 10.1080/15592294.2016.1144006

**Published:** 2016-02-18

**Authors:** Batbayar Khulan, Lincoln Liu, Catherine M. Rose, Ashley K. Boyle, Jonathan R. Manning, Amanda J. Drake

**Affiliations:** University/BHF Center for Cardiovascular Science, University of Edinburgh, Queen's Medical Research Institute, 47 Little France Crescent, Edinburgh, UK

**Keywords:** DNA methylation, glucocorticoids, heme, liver, prenatal

## Abstract

Glucocorticoids are widely used in threatened preterm labor to promote maturation in many organ systems in preterm babies and have significant beneficial effects on morbidity and mortality. We performed transcriptional profiling in fetal liver in a rat model of prenatal glucocorticoid exposure and identified marked gene expression changes in heme biosynthesis, utilization, and degradation pathways in late gestation. These changes in gene expression associated with alterations in DNA methylation and with a reduction in hepatic heme concentration. There were no persistent differences in gene expression, DNA methylation, or heme concentrations at 4 weeks of age, suggesting that these are transient effects. Our findings are consistent with glucocorticoid-induced accelerated maturation of the haematopoietic system and support the hypothesis that glucocorticoids can drive changes in gene expression in association with alterations in DNA methylation.

## Introduction

The ability of glucocorticoids to function as promoters of maturation in organ systems is widely exploited pharmacologically as a treatment for women with threatened preterm labor with significant beneficial effects on morbidity and mortality in babies born preterm.^1^ The major reason underpinning the use of antenatal glucocorticoids is to enhance lung maturation,^1^ and glucocorticoids induce the expression of genes involved in many processes in the developing lung, including the synthesis of surfactant proteins.^2,3^ However, glucocorticoids are also known to stimulate maturation in many other differentiating tissues, including the liver, pancreas, kidney, and heart.^4^

In order to understand the mechanisms by which glucocorticoids affect fetal development and maturation, a number of animal models have been developed.^5^ Using one such model, in which pregnant rat dams are treated with the synthetic glucocorticoid Dexamethasone (Dex) during the last week of pregnancy, we have previously shown that prenatal glucocorticoid overexposure alters the expression of candidate genes in fetal liver.^6-8^ In this study, we set out to identify additional pathways that are affected by prenatal glucocorticoid exposure by performing transcriptional profiling in late gestation fetal liver at embryonic day (e)20 from prenatally glucocorticoid-exposed rats. To determine whether changes were persistent, we also analyzed candidate gene expression in liver at 4 weeks of age. Since a growing number of studies, including in this model, suggest that early life exposure to glucocorticoid excess may drive changes in gene expression through alterations in the epigenome, particularly DNA methylation changes,^6,9,10^ we additionally performed analysis of DNA methylation at promoters of differentially expressed candidate genes.

## Results

### Dex-exposed males exhibit transcriptional changes in the liver heme pathway at e20

**Figure 1.**
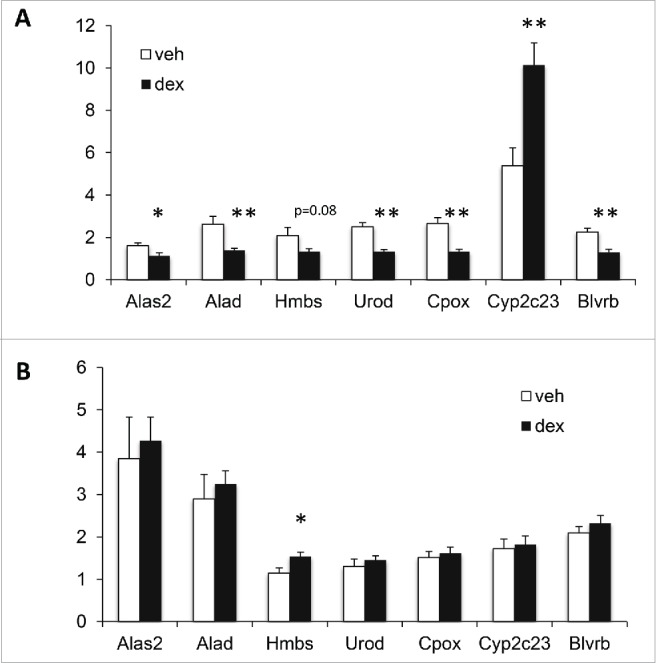
Prenatal glucocorticoid overexposure is associated with altered expression of genes in the heme pathway at embryonic day (e)20 but not at 4 weeks. A) Gene expression at e20. B) Gene expression at 4 weeks. n=8 per group at each time point. Data are mean ± SEM. *P < 0.05, **P < 0.01.

A total of 134 genes were differentially expressed between Dex and Veh male liver (*P* < 0.01 and a difference of 10% or greater). Pathway analysis using the GeneGo tool revealed marked gene expression changes in heme biosynthesis, utilization, and degradation pathways (Supplementary Fig. 1A and B). Quantitative PCR validation confirmed that Dex exposure was associated with decreased expression of 4 genes involved in the heme biosynthesis pathway (*Alad*: 1.9-fold, *P* = 0.005, *Cpox*: 2-fold, *P* = 0.0006, and *Urod*: 1.9-fold, *P* = 0.0001, and the rate-limiting enzyme *Alas2*: 1.4-fold, *P* = 0.03) (Fig. 1A). Dex exposure also decreased the expression of Biliverdin reductase B (*Blvrb*), which is involved in heme degradation (1.7 fold, *P* = 0.001) (Fig. 1A). In contrast, Dex exposure was associated with increased expression of mRNA encoding the heme-containing cytochrome P450 2C23 enzyme (*Cyp2c23*: 1.9-fold, *P* = 0.003) (Fig. 1A). The experiment was not designed to analyze the changes in gene expression over time so we are unable to compare pre- and post-natal gene expression directly. However, none of the expression changes identified at e20 was maintained at 4 weeks of age, although we identified increased expression of Hmbs postnatally (Fig. 1B).

### DNA methylation changes in alternative promoters of Hmbs and Alad may facilitate expression differences at e20

**Figure 2.**
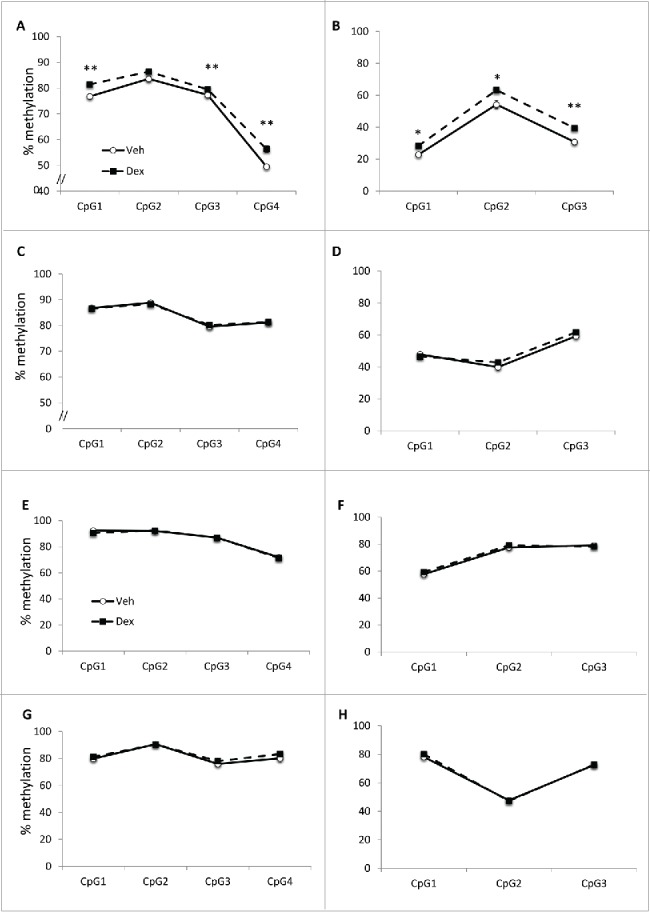
DNA methylation at *Alad* and *Hmbs*. Pyrosequencing analysis showed increased DNA methylation at the erythroid promoter of (A) *Alad* and (B) *Hmbs* in Dex-exposed fetuses, and absence of any differences in DNA methylation in gene bodies of (C) *Alad* and (D) *Hmbs* gene bodies. At 4 weeks, there were no persistent differences in DNA methylation at the erythroid promoters of (E) *Alad* and (F) *Hmbs* or in the gene bodies of (G) *Alad* or (H) *Hmbs*. Data are mean ± SEM. *P < 0.05, **P < 0.01.

Two genes in the heme synthesis pathway, *Alad* and *Hmbs*, have erythroid-specific and housekeeping isoforms, the expression of which are driven from different promoters. Given the changes in *Alad* gene expression at e20, we proceeded to analyze DNA methylation at the alternate promoters of both genes. The housekeeping promoters for both *Alad* and *Hmbs* are constitutively unmethylated, and there was no difference in methylation levels between the two groups (Table 2). In contrast, at e20, DNA methylation was significantly increased in Dex-exposed animals at the erythroid-specific promoter of both genes (Fig. 2A and B). There were no differences in DNA methylation in the gene body (Fig. 2C and D). There were no persistent differences in DNA methylation at 4 weeks (Fig. 2E-H).

**Table 2.** DNA methylation at the housekeeping promoters of Alad and Hmb*s.*

		CpG1	CpG2	CpG3	CpG4
Alad	Veh	0.78 ± 0.16	0.93 ± 0.19	0.69 ± 0.13	1.36 ± 0.21
	Dex	0.78 ± 0.18	0.94 ± 0.19	0.72 ± 0.14	1.08 ± 0.20
Hmbs	Veh	0.53 ± 0.03	0.44 ± 0.07	0.61 ± 0.03	0.52 ± 0.03
	Dex	0.47 ± 0.04	0.26 ± 0.07	0.64 ± 0.03	0.46 ± 0.02

Data are expressed as % methylation ± SEM

### Dex exposure is associated with DNA methylation changes in the Cyp2c23 promoter and gene body

**Figure 3.**
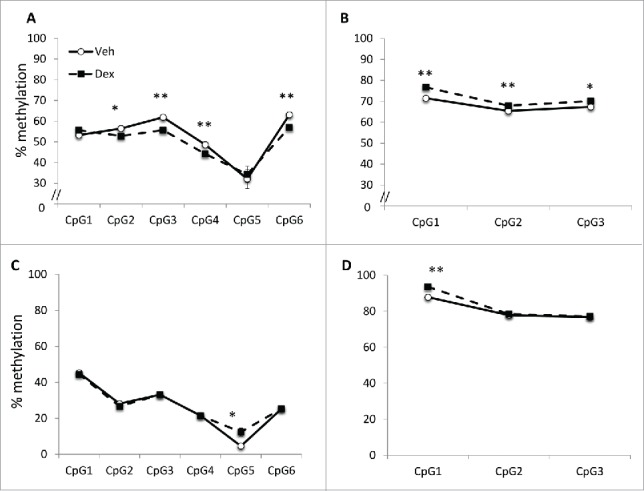
DNA methylation at *Cyp2c23*. Pyrosequencing analysis showed decreased DNA methylation in the (A) *Cyp2c23* promoter and increased methylation in the (B) *Cyp2c23* gene body in Dex-exposed fetuses. At 4 weeks, DNA methylation was increased at a single CpG in the (C) *Cyp2c23* promoter and (D) *Cyp2c23* gene body in Dex-exposed fetuses. Data are mean ± SEM. *P < 0.05, **P < 0.01.

Given the increase in Cyp2c23 expression at e20 we proceeded to investigate DNA methylation at the *Cyp2c23* promoter and gene body. At e20, Dex exposure was associated with decreased DNA methylation at the *Cyp2c23* promoter (4 CpGs out of 6 tested), but with increased DNA methylation in the *Cyp2c23* gene body (Fig. 3A and B). DNA methylation at the *Cyp2c23* gene promoter decreased between e20 and 4 weeks of age and, although DNA methylation differences present at e20 were not persistent, a new difference emerged at a single CpG at 4 weeks (Fig. 3C). In contrast, DNA methylation increased in the *Cyp2c23* gene body over time and there was a persistent difference in DNA methylation at a single CpG (Fig. 3D).

### Dex exposure decreases hepatic heme concentrations at e20

In order to identify potential functional consequences of alterations in gene expression and DNA methylation changes in genes important in the heme pathway, we quantified heme concentrations in the liver. At e20, Dex exposure reduced liver heme protein content by 1.6-fold (Veh 26.7 ± 4.2 vs. Dex 16.6 ± 1.7 μM/mg; *P* = 0.048). While liver heme protein content was reduced in the postnatal samples compared to e20, there were no persistent differences between groups (Veh 10.1±0.6 vs. Dex 9.4 ± 0.4 μM/mg).

## Discussion

Here we have shown that prenatal glucocorticoids impact the heme pathway in fetal liver at multiple levels with effects on gene transcription, DNA methylation, and hepatic heme content. During mammalian development, hematopoiesis initiates at multiple times and locations: in early development, hematopoiesis occurs in the yolk sac, allantois, placenta, and aorta–gonad–mesonephros, followed by a shift to fetal liver (from day e11–12 in mice and by 5 weeks post-conception in humans).^11^ In mice, hematopoiesis begins to occur in the spleen from day e14 and in the bone marrow from e18. In humans, hematopoiesis moves from the fetal liver to the bone marrow at ∼12 weeks post-conception. Although in postnatal life most heme synthesis occurs in developing red cells in the bone marrow, the formation of heme-containing enzymes, including the cytochrome P450 enzymes, persists in the liver.^12^ In our study, expression microarray and qPCR analysis of gene expression at e20 revealed that prenatal Dex was associated with decreased expression of genes involved in heme synthesis in rat liver. These results suggest that the transition from liver to bone marrow hematopoiesis is accelerated with Dex treatment; this is supported by our findings of lower hepatic heme following Dex exposure. These changes appear to be transient, however, since there were no persistent differences in gene expression or hepatic heme concentrations at 4 weeks of age.

Two of the genes involved in heme synthesis, *Alad* and *Hmbs*, utilize alternative promoters allowing erythroid-specific and non-erythroid regulation.^12^ Transcription of the full-length isoforms is regulated by housekeeping promoters, which overlap CpG islands and are expressed in all tissues, whereas tissue-specific promoters regulate transcription of the shorter isoforms, which are expressed only in erythroid cells.^13,14^ As expected, given that they overlap CpG islands, the housekeeping promoters of *Alad* and *Hmbs* were unmethylated. In contrast, the erythroid cell specific promoter of *Alad* showed modest but significant differences in DNA methylation, which correlated with the observed changes in gene expression. Although there were no significant changes in Hmbs expression, we also observed an increase in DNA methylation at the erythroid cell specific promoter, suggesting a shift away from erythroid-specific expression. These differences in DNA methylation were not persistent at four weeks of age, at which time DNA methylation levels were much higher than at e20, consistent with the postnatal silencing of erythroid cell specific promoter driven gene expression.

The peak expression of Cyp2c23 occurs around the time of birth ^15,16^ so that the Dex-induced increase in expression at e20 again suggests premature maturation. Since heme is required for incorporation into cytochrome enzymes, including Cyp2c23 in hepatocytes, increased utilization could be an additional explanation for the decreased hepatic heme concentrations at e20. The decrease in *Cyp2c23* promoter DNA methylation and increased methylation in the gene body are consistent with the observed increase in gene expression. In contrast to the lack of DNA methylation changes at *Alad* and *Hmbs* at 4 weeks of age, there was a persistent difference in DNA methylation at a single CpG in the *Cyp2c23* promoter at 4 weeks and a new difference emerged at one CpG in the *Cyp2c23* gene body. Nevertheless, the significance of these changes at 4 weeks is unclear since there was no difference in gene expression at this time.

Our data for the *Cyp2c23* promoter, suggesting that glucocorticoids associate with alterations in DNA methylation that may facilitate gene transcription are in agreement with previous studies showing that glucocorticoids induce demethylation of the hepatic tyrosine aminotransferase gene promoter in late gestation, which is permissive for transcription factor binding ^17^ and the induction of transcription in early postnatal life in response to hypoglycemia.^18^ However, glucocorticoids both stimulate and repress gene transcription,^19^ so that both decreases and increases in DNA methylation might be expected to occur as a consequence of glucocorticoid exposure. Thus, in contrast to the decrease in DNA methylation observed at the *Cyp2c23* promoter, increased methylation was observed in association with repression of transcription, most notably at the erythroid-specific promoter of *Alad*. Although the changes in DNA methylation were modest, they are consistent with other studies showing effects of the prenatal environment.^20^ Additionally, for *Alad* and *Hmbs*, the normal increase in DNA methylation at the erythroid-specific promoters between e20 and 4 weeks further supports the suggestion that glucocorticoids induce early maturation of this pathway.

Although the main reason for antenatal glucocorticoid use in the context of threatened preterm labor is for enhancement of fetal lung maturation,^1^ glucocorticoids also stimulate maturation in other tissues ^4^ and our study provides further evidence for effects on the fetal liver. At around the time of birth, the liver switches from an organ involved in hematopoiesis to one primarily involved in metabolism, with functions including detoxification, regulation of glycogen storage and protein synthesis. Previous studies in the model used here have shown that prenatal glucocorticoid exposure stimulates the premature production of the hepatic gluconeogenic enzyme phosphoenolpyruvate carboxykinase, erythropoietin, and the transcription factor HNF4α.^7^ Our data, suggesting that glucocorticoids also promote early maturation of hematopoiesis, is consistent with data from primary fetal hepatic cell culture, in which glucocorticoids are associated with suppression of *in vitro* hematopoiesis.^21^ Taken together, these results suggest that glucocorticoid-induced fetal hepatic maturation is associated with the termination of hematopoiesis and the relocation of haematopoietic cells. These changes may be beneficial in the context of preterm birth, promoting maturation of metabolic function and facilitating adaptation to the extrauterine environment.

There are, however, ongoing concerns about the long-term effects of prenatal glucocorticoid overexposure, particularly with repeated dosing.^1^ Our data suggest that the majority of glucocorticoid-induced changes in gene transcription and DNA methylation in relation to the heme pathway are not persistent, at least up to 4 weeks of age. Nevertheless, there were some persistent changes in DNA methylation in the *Cyp2c23* promoter and gene body. Cyp2c enzymes epoxidise arachidonic acid to metabolites involved in the regulation of vascular and renal function ^22^ and, since prenatal glucocorticoid overexposure is associated with the development of hypertension in adulthood,^23^ persistent effects on *Cyp2c23* expression or DNA methylation may deserve further investigation. Although these persistent changes were CpG specific, previous studies have suggested that site-specific differences in DNA methylation may be important in mediating changes in the postnatal phenotype.^20,24,25^

In conclusion, our data showing that prenatal glucocorticoids induce changes in gene expression and DNA methylation at key genes in the heme biosynthesis pathway suggest a mechanism through which glucocorticoids associate with accelerated maturation.

## Material and methods

### Animals and tissues

Pregnant female Wistar rats were injected subcutaneously with either Dex (100 mcg/kg in 0.9% saline containing 4% ethanol) or with equivalent volume of vehicle (Veh; 0.9% saline containing 4% ethanol) administered daily between 0800 and 0900 from e15 to e21 inclusively. Our previous studies in this model have shown that this dose of Dex reduces birth weight without affecting litter size or gestation length.^26^ A subgroup of pregnant females were killed at e20 (8 Dex and 8 Veh). Males were identified by visual inspection (ano-genital distance) and this was then confirmed by PCR for the SRY gene (forward primer ATC TTC AAG GCG CTG CAA; reverse primer CGG TGG ACC CTG AGA TTG). Male fetal liver samples were collected and snap-frozen on dry ice and stored at −80°C. All remaining females (5 Dex and 7 Veh) were allowed to deliver their pups naturally between e21.5 and e23.5. Litters were killed back to 8 per litter, retaining similar numbers of both sexes. At 28 days, males were sacrificed by decapitation following CO_2_ asphyxiation. Liver was harvested, snap frozen on dry ice, and stored at −80°C.

### Extraction of DNA and RNA samples

Genomic DNA and total RNA samples were extracted using Qiagen DNeasy and RNeasy kits (Qiagen, Crawley, UK) following the manufacturer's instructions. Both DNA and RNA samples were quantified using Qubit (Life Technologies Ltd, Paisley, UK) and the integrity of DNA and RNA samples were analyzed by gel electrophoresis and Agilent Bioanalyzer (Agilent Technologies, Santa Clara, CA, USA).

### Illumina RatRef-12 expression BeadChip array

For 3 males per group from separate litters at e20, we undertook microarray profiling of gene expression in fetal liver. RNA labeling was performed on RNA (500 ng) using the Illumina® Total Prep RNA amplification kit (Life Technologies, Paisley, UK) and subsequently hybridized to Illumina RatRef-12 expression BeadChip arrays as per the manufacturer's instructions at the Wellcome Trust Clinical Research Facility, Genetics Core, Western General Hospital, Edinburgh, UK.

### Expression analysis by RT qPCR

RNA (500 ng) was reverse transcribed for both e20 and 4 weeks postnatal samples (e20: 8 biological replicates per group from 8 Dex and 8 Veh litters; 4 weeks: 6 biological replicates per group from 5 Dex and 6 Veh litters) using the Promega Reverse Transcription kit (Promega, Southampton, UK) and real time PCR was performed using the UPL system from Roche Diagnostics Ltd., using a Roche Lightcycler 480 (Roche, West Sussex, UK). Primers were designed using Roche Universal Probe Library Assays (Roche, West Sussex, UK) and details are given in Table 1. Gene expression was normalized to the expression of *GAPDH* (e20) or the mean of *Pgk1* and *YWHAZ* (4 weeks).

**Table 1.** Primer details for qPCR and pyrosequencing analysis.

qPCR analysis			
Gene	Forward	Reverse	UPL probe
Alas2	caggggctttcctgttatcc	tgttgagtgccgcattacc	5
Cpox	gtcctgaagcacacaggtga	tctgccctctggttttctgt	10
Urod	ttagcaatgtagcgctgtgg	tctaggaagagattggtcgactg	84
Alad	gcctttgatctcaggactgc	ggggtgcaaagtaggtgatg	109
Hmbs	tccctgaaggatgtgcctac	acaagggttttcccgtttg	79
Cyp2c23	ccctcgggactacattgact	gatggaactcagacttcaggttg	63
Pyrosequencing			
Gene	Forward	Reverse	Sequencing
Alad P1	gtttagaagggagtgtaggttgta	ctaccaaaaaccctactcaccacc	ggagtgtaggttgtatttt
Alad P2	ttgagatagggttggttttgaatt	ccccacaaaaactctataactaacc	ggatgattatggatttttga
Alad GB	ataagtggaagtttggggaaat	attaatacactcaccatcctaatca	caaaaacaaacaaattaaacaatat
Hmbs P1	ggttttttggagtttgtagaag	aactcccaccccatataccttcaat	tttggagtttgtagaagt
Hmbs P2	tgagtgggagggttgtata	atctatcctaccccaacctct	aatgataaggtttattagttttaag
Hmbs GB	aggtagaataagtgggaagtagaa	tcaataccattatcctaactataactaacc	gggaagtagaataggg
Cyp2c23 P1	aggggaagtattttttgtataggtat	tccccactttaaaacacattccttatt	agtattttttgtataggtatgtt
Cyp2c23 P2	agggttaaaatggagttgttgg	ccccctaatacccaattttatccacacta	atggagttgttggga
Cyp2c23 GB	gttagggttttgtagtgttttaagt	tttccctatatcattaaaatctttct	ttggataaattattatatttttttg

### DNA methylation analysis by bisulfite pyrosequencing

Genomic DNA (1μg) was subjected to bisulfite treatment using the EZ-DNA methylation kit (Zymo Research, Irvine, CA, USA) for both e20 and 4 weeks postnatal samples (e20: 8 biological replicates per group from 8 Dex and 8 Veh litters; 4 weeks: 8 biological replicates per group from 5 Dex and 7 Veh litters). Loci of interest were amplified using Pyrosequencing primers (Table 1) designed using PyroMark Assay design 2.0 software (Qiagen, Crawley, UK). Data were analyzed using PyroMark 24 software (Qiagen, Crawley, UK).

### Heme measurement

Total liver heme was quantified using a QuantiChromTM Heme Assay kit (DIHM-250) (BioAssay Systems, CA, USA) for both e20 and 4 weeks postnatal samples (e20: 8 biological replicates per group from 8 Dex and 8 Veh litters; 4 weeks: 8 biological replicates per group from 5 Dex and 7 Veh litters). About 30 mg of liver was homogenized in 500 ml of buffer containing 100 mM K_2_HPO_4_ pH7.4 and 2 mM MgCl_2_. Five times diluted homogenate was used for measuring heme, following the manufacturer's protocol. The values were normalized to total protein content measured using the Bradford assay (BIO-RAD, Hemel Hempstead, UK).

### Data and statistical analysis

Microarray data were read and processed with the Bioconductor suite.^27^ The Lumi package ^28^ was used for preprocessing, applying a background correction based on control probes prior to quantile normalization. Differential expression was assessed with a combination of fold change and a rank product false discovery rate.^29^ Gene expression, site-specific DNA methylation and heme concentrations were analyzed by independent Student t testing. Data are expressed as mean ± SEM.

## Supplementary Material

KEPI_A_1144006_s02.zip
