# Supplementary material for: Glucocorticoids accelerate maturation of the heme pathway in fetal liver through effects on transcription and DNA methylation
Source: Epigenetics. 2016 Feb 18;11(2):103–9. doi: 10.1080/15592294.2016.1144006 (PMC4846099; doi:10.1080/15592294.2016.1144006)
Supplement: KEPI_A_1144006_s02.zip [file kepi-11-02-1144006-s001.zip › KEPI_A_1144006_s02 captions.docx]

**Supplementary Figure 1:**

(A) Schematic diagram of the heme pathway with the location of genes discussed in this study indicated. (B) Pathway analysis using the GeneGo tool revealing marked gene expression changes in heme biosynthesis, utilization, and degradation pathways.
