# Supplementary material for: Glucocorticoids accelerate maturation of the heme pathway in fetal liver through effects on transcription and DNA methylation
Source: Epigenetics. 2016 Feb 18;11(2):103–9. doi: 10.1080/15592294.2016.1144006 (PMC4846099; doi:10.1080/15592294.2016.1144006)
Supplement: KEPI_A_1144006_s02.zip [file kepi-11-02-1144006-s001.zip › KEPI_A_1144006_s02.pptx]

## Slide 1
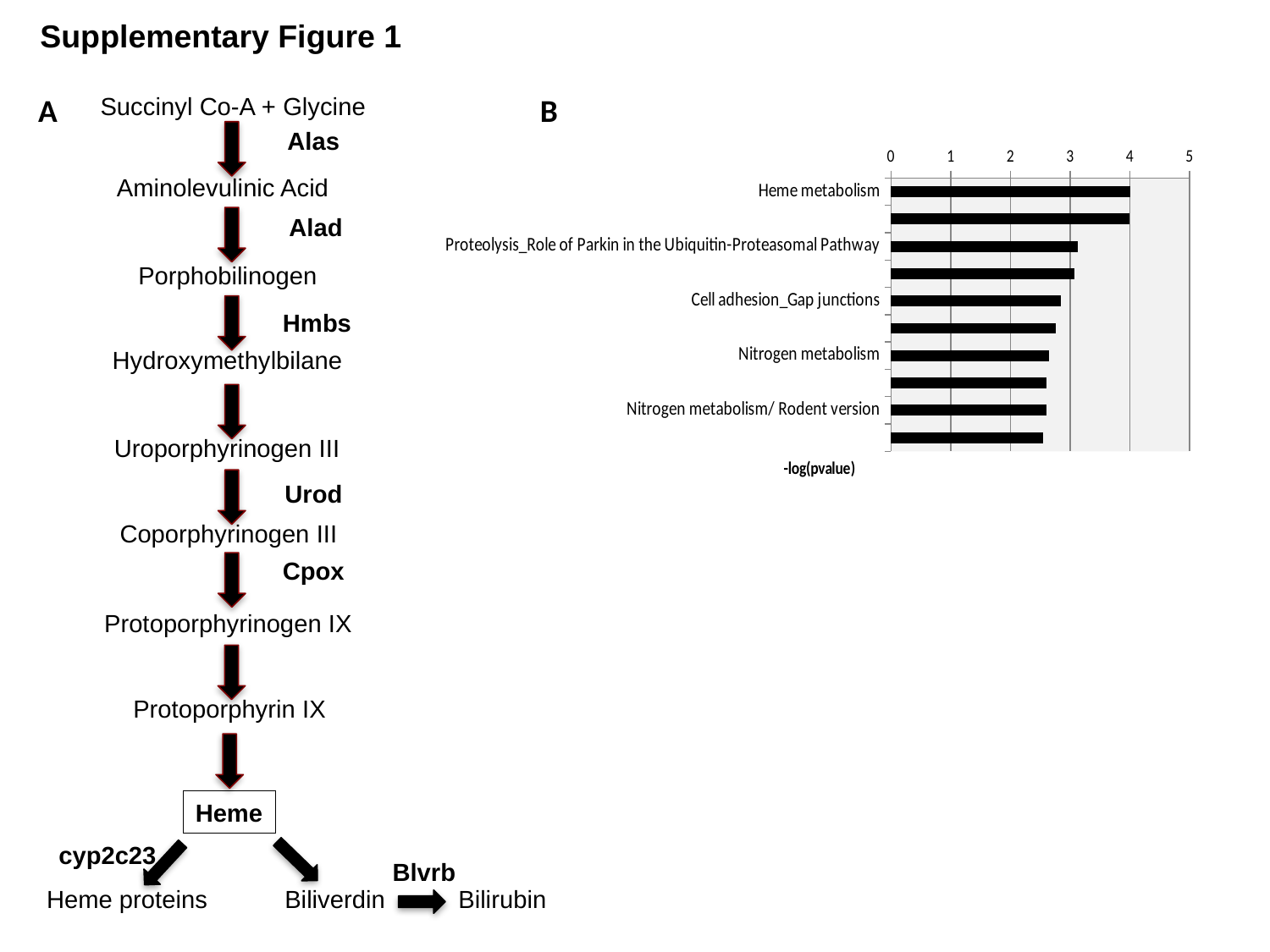

Supplementary Figure 1
### Chart
| Category | |
|---|---|
| Heme metabolism | 4.001304841688344 |
| Cell cycle_Spindle assembly and chromosome separation | 3.98927613460819 |
| Proteolysis_Role of Parkin in the Ubiquitin-Proteasomal Pathway | 3.1298888446356 |
| Cytoskeleton remodeling_Neurofilaments | 3.076704159344496 |
| Cell adhesion_Gap junctions | 2.84163750790475 |
| Cell cycle_Role of Nek in cell cycle regulation | 2.75920122888267 |
| Nitrogen metabolism | 2.6458915608526 |
| Cytoskeleton remodeling_Keratin filaments | 2.610479534153617 |
| Nitrogen metabolism/ Rodent version | 2.610479534153617 |
| Transcription_Role of AP-1 in regulation of cellular metabolism | 2.542572307053515 |A
Succinyl Co-A + Glycine
Alas
Alad
Hmbs
Urod
Cpox
Aminolevulinic Acid
Porphobilinogen
Hydroxymethylbilane
Uroporphyrinogen III
Coporphyrinogen III
Protoporphyrinogen IX
Protoporphyrin IX
Heme
cyp2c23
Blvrb
Heme proteins
Biliverdin
Bilirubin
B
